# Supplementary figures and images for: The SRG rat, a Sprague-Dawley Rag2/Il2rg double-knockout validated for human tumor oncology studies
Source: PLoS One. 2020 Oct 7;15(10):e0240169. doi: 10.1371/journal.pone.0240169 (PMC7540894; doi:10.1371/journal.pone.0240169)

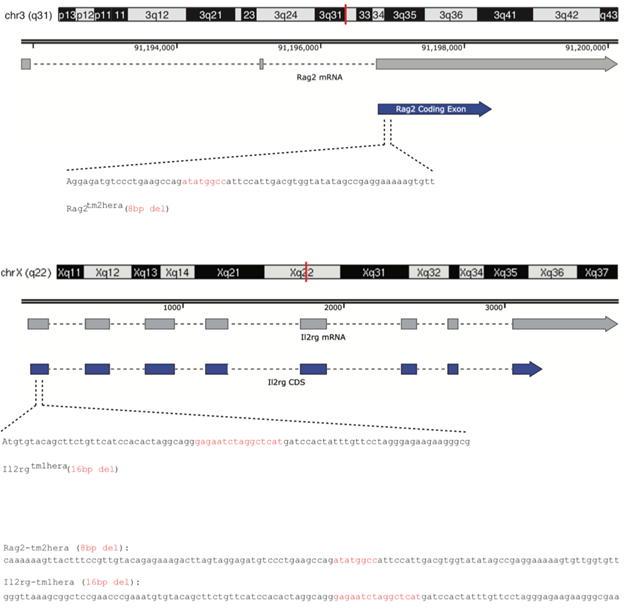

Supplement: S1 Fig — The SRG rat also carries a 16bp deletion in the first exon of the Il2rg gene to knock out its function. (TIF) [file pone.0240169.s001.tif]

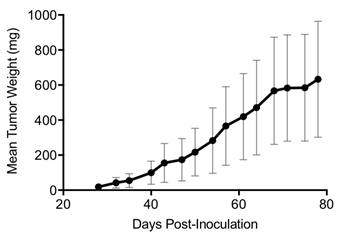

Supplement: S2 Fig — Mean weight in mg with SEM. (TIF) [file pone.0240169.s002.tif]

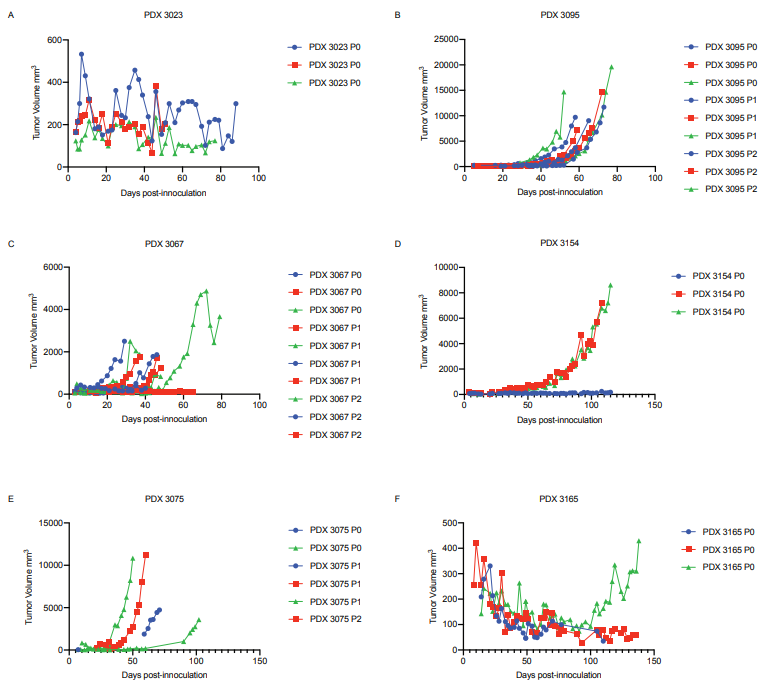

Supplement: S3 Fig — Each graph depicts tumor volumes for individual animals for 6 different NSCLC patient samples (A-F). P1 is the inital implant into animals using fresh patient tissue, P2 is the first serial passage from animal to animal, P3 is the second serial passage from animal to animal. Sample 3067 (C) was implanted into SRG rats for P1 and then serially implanted into NSG mice for P2 and P3. (TIFF) [file pone.0240169.s003.tiff]

Figure 4B

AR

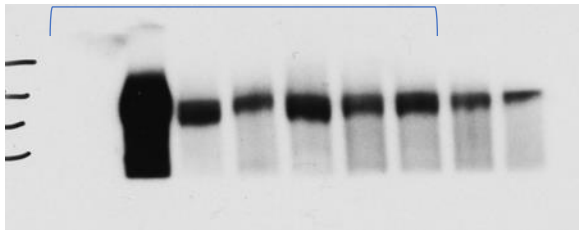

GAPDH

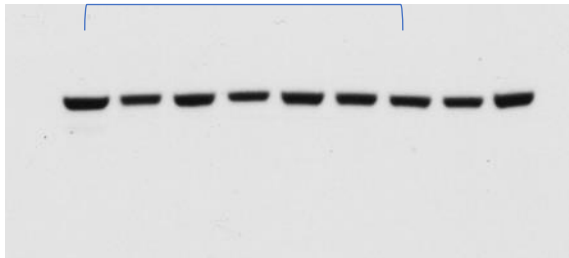

Figure 4C

AR

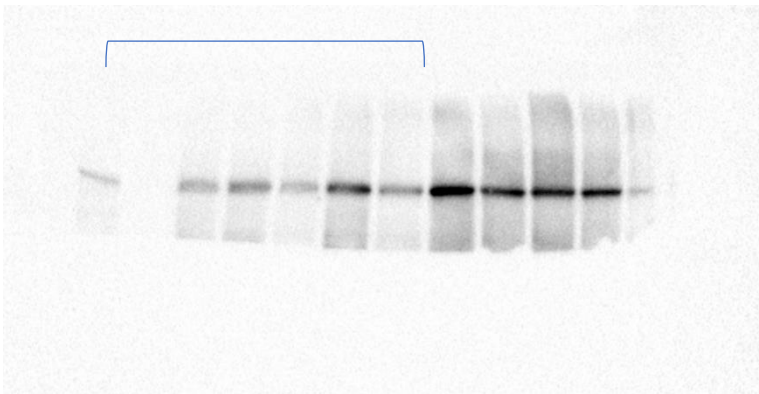

GAPDH

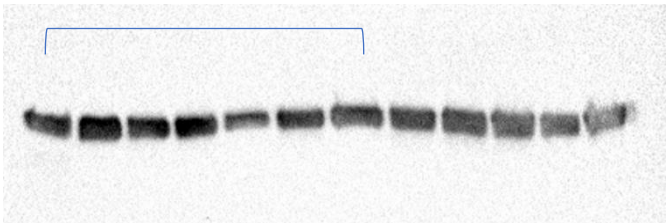

Supplement: S4 Fig — (PDF) [file pone.0240169.s004.pdf]
